# Understanding the seasonality of performance resilience to climate volatility in Mediterranean dairy sheep

**DOI:** 10.1038/s41598-021-81461-8

**Published:** 2021-01-21

**Authors:** Valentina Tsartsianidou, Vanessa Varvara Kapsona, Enrique Sánchez-Molano, Zoitsa Basdagianni, Maria Jesús Carabaño, Dimitrios Chatziplis, Georgios Arsenos, Alexandros Triantafyllidis, Georgios Banos

**Affiliations:** 1 Department of Genetics, Development & Molecular Biology, School of Biology, Aristotle University of Thessaloniki, 54124 Thessaloniki, Greece; 2 Department of Animal and Veterinary Sciences, Scotland’s Rural College, Roslin Institute Building, Easter Bush, Midlothian, EH25 9RG UK; 3 Division of Genetics and Genomics, The Roslin Institute and Royal (Dick) School of Veterinary Studies, University of Edinburgh, Easter Bush, Midlothian, EH25 9RG UK; 4 Department of Animal Production, School of Agriculture, Aristotle University of Thessaloniki, 54124 Thessaloniki, Greece; 5 Departamento de Mejora Genética Animal, Instituto Nacional de Investigación y Tecnología Agraria y Alimentaria (INIA), 28040 Madrid, Spain; 6 Laboratory of Agrobiotechnology and Inspection of Agricultural Products, Department of Agriculture, International Hellenic University, Alexander Campus, 57400 Sindos, Greece; 7 Laboratory of Animal Husbandry, School of Veterinary Medicine, Aristotle University of Thessaloniki, 54124 Thessaloniki, Greece

**Keywords:** Computational biology and bioinformatics, Ecology, Genetics

## Abstract

As future climate challenges become increasingly evident, enhancing performance resilience of farm animals may contribute to mitigation against adverse weather and seasonal variation, and underpin livestock farming sustainability. In the present study, we develop novel seasonal resilience phenotypes reflecting milk production changes to fluctuating weather. We evaluate the impact of calendar season (autumn, winter and spring) on animal performance resilience by analysing 420,534 milk records of 36,908 milking ewes of the Chios breed together with relevant meteorological data from eastern Mediterranean. We reveal substantial seasonal effects on resilience and significant heritable trait variation (h^2^ = 0.03–0.17). Resilience to cold weather (10 °C) of animals that start producing milk in spring was under different genetic control compared to autumn and winter as exemplified by negative genetic correlations (− 0.09 to − 0.27). Animal resilience to hot weather (25 °C) was partially under the same genetic control with genetic correlations between seasons ranging from 0.43 to 0.86. We report both favourable and antagonistic associations between animal resilience and lifetime milk production, depending on calendar season and the desirable direction of genetic selection. Concluding, we emphasise on seasonal adaptation of animals to climate and the need to incorporate the novel seasonal traits in future selective breeding programmes.

## Introduction

Global climate change affects agricultural production and raises major concerns about future availability of plant and animal products. The direct and indirect impact on livestock production of increasing air temperature, frequency of extreme weather events, and seasonal and inter-annual weather variability differs by geographic region, animal species and production type^1,2^. The Mediterranean basin is considered as one of the most vulnerable regions for climate change^3^, which challenges the productive performance of livestock raised there^4–6^. According to climate change projections for the Mediterranean region, in addition to average air temperature, the inter-annual variability is predicted to increase, and precipitation levels are expected to decrease mostly during summer^7,8^.

The impact of climate alterations on seasonal variability, such as frequency of extreme weather events and changing conditions, raises concerns regarding the performance and productivity of livestock species. For example, seasonality influences many physiological mechanisms of small ruminants (sheep, goats), due to changing daylight length, air temperature and precipitation^9^. Typically, seasonal effects on small ruminant milk performance have been attributed to temperature and photoperiod variation throughout the year^10^. Additionally, animals usually regulate their reproduction according to the seasonal environmental variation^9^. Further changes to the already established seasonal rules will challenge animal response and adaptation and, consequently, impact on their performance.

The intensification of livestock production systems has resulted in highly productive animals, which are genetically more susceptible to environmental stressors such as disease or climatic fluctuations^11,12^. Consequently, animal fitness and environmental adaptability are continuously compromised. Furthermore, livestock diseases are highly affected by climate change due to exposure of animals to increased temperature and humidity^13^. Reduced fertility and increased mortality have been also reported in livestock species under intensive selection^14^. Large energy amounts spent by an animal on production may lead to reduced available energy for other physiological processes including maintenance, growth, immune response and reproduction. Notably, an environment-dependent homeostasis threshold is known to be affected by reduction of energy supporting the latter processes, which may increase in demanding environments^15^.

Therefore, it is beneficial in many aspects to enhance the animals’ capacity to remain unaffected from environmental (including climate) variation and disturbances and maintain their normal levels of production and other physiological processes. We term this capacity as animal resilience according to Colditz and Hine^16^. The enhancement of individual animal resilience through selective breeding may contribute to mitigation against changing climate and increasing weather variability^17,18^.

However, the complexity of animal resilience implies difficulties in trait definition and measurement^19–24^, and implementation in selective breeding programmes^25^. Recently, several studies have been conducted on Mediterranean dairy ruminant resilience to heat stress^26–28^ and the derivation of novel phenotypes for small ruminant resilience to weather variability^29^; applying the reaction norm approach. Reaction norm functions fitted in random regression models have been considered in the study of the interaction between livestock and environment, including performance resilience to climate change^30–32^. In such analyses, individual animal phenotypes are characterised as ‘function-valued’ traits that change continuously in response to an environmental variable^33^.

Although animal performance resilience is receiving increasing emphasis in research studies and the international scientific literature, the impact of seasonal effects on resilience traits has not been examined to our knowledge. Seasonal climate conditions are known to affect milk production of ruminants^34,35^. In the present study we hypothesise that this effect extends to milk production changes in response to weather volatility, whose profile also differs across calendar seasons.

Our specific objectives were to (i) assess the impact of calendar season on animal performance resilience, (ii) develop novel seasonal resilience phenotypes in Chios dairy sheep reflecting changes in milk yield in response to climatic fluctuations and (iii) estimate genetic parameters for the novel seasonal resilience phenotypes and milk production. Reaction norms were deployed to derive seasonal performance resilience phenotypes and mixed models were implemented to estimate the genetic parameters.

## Results

### Data descriptive statistics

Descriptive statistics of animal performance and weather variables are presented in Table 1 by lambing season. Average lifetime milk yield, number of days milked and number of lactations decreased as the calendar season of lambing progressed from autumn to spring. This decrease was quite similar between lifetime milk produced and length of productive life and, therefore, average test-day milk yield remained relatively constant across the three seasons. The respective weather statistics corresponding to the day of milk measurement were nearly identical to those during the week preceding the milk measurement.

**Table 1:** Descriptive statistics (mean and SD in parentheses) of animal performance and weather variables by lambing season.

	Autumn (17,899 animals)	Winter (24,837 animals)	Spring (8363 animals)
Test-day milk yield (kg)	1.52 (0.75)	1.42 (0.73)	1.45 (0.77)
Lifetime milk yield (kg)	573.74 (460.39)	373.17 (269.77)	225.23 (222.43)
Total DIM	374.88 (254.54)	260.24 (166.13)	154.31 (116.45)
Total lact	2.13 (1.33)	1.68 (0.93)	1.24 (0.60)
Tavg (°C)	13.44 (8.02)	19.50 (7.41)	23.81 (5.57)
RHavg (%)	68.29 (15.61)	62.42 (14.38)	59.40 (13.75)
THIavg	13.98 (4.16)	17.76 (5.59)	19.63 (3.17)

Total DIM: Total number of days milked, Total lact: Total number of lactations milked, Tavg: average air temperature, RHavg: average relative humidity, THIavg: average temperature-humidity index.

Results from the analyses involving the two weather variables, air temperature and THI, were very similar to each other. This is mainly attributable to the limited variation observed in relative humidity compared to air temperature in our data. Thus, variation in THI essentially reflected variation in air temperature in the present study. Therefore, the remaining of the manuscript focuses only on air temperature. Monthly variation of the latter in the data during the period of the study is illustrated in Supplementary Fig. S1.

### Reaction norms at population level

**Figure 1:**
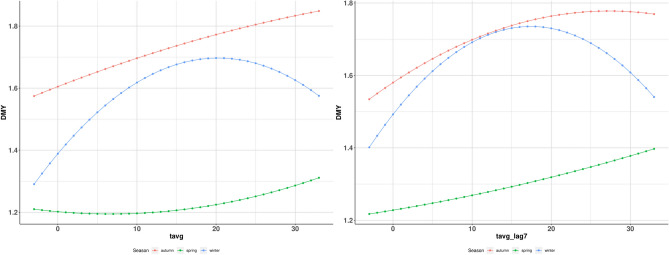
Derived average reaction norm by lambing season from the Eq. (2) corresponding to population level response. Each curve represents changes in daily milk yield (DMY, kg) in response to average air temperature variation on the milk test date (tavg, °C) and cumulatively during the week preceding the milk test date (tavg_lag7, °C), respectively. Red line: autumn lambing, blue line: winter lambing, green line: spring lambing.

Figure 1 illustrates the population level milk yield change across the air temperature gradient on the date of milk test and the week average preceding the milk test. Different population responses were observed in the three seasons of study, indicating a seasonal resilience of Chios milk performance to temperature fluctuations. Winter lambing ewes showed the most variable performance change in response to temperature fluctuation. While, average milk yield responded positively to increasing temperature up to 20 °C , it notably decreased when temperature exceeded this value, potentially indicating compromised performance due to heat stress. Conversely, milk yield of autumn and spring lambing ewes reacted positively to increasing air temperature throughout the temperature range. Minor differences were observed between animal response to daily and cumulative (preceding week) air temperature variation. Winter lambing animals in particular were more prone to the cumulative than the daily temperature effect under heat stress conditions. Furthermore, spring lambing ewes demonstrated a more linear relationship of their milk yield with rising cumulative than daily temperature (Fig. 1).

### Resilience phenotypes at individual animal level

**Figure 2:**
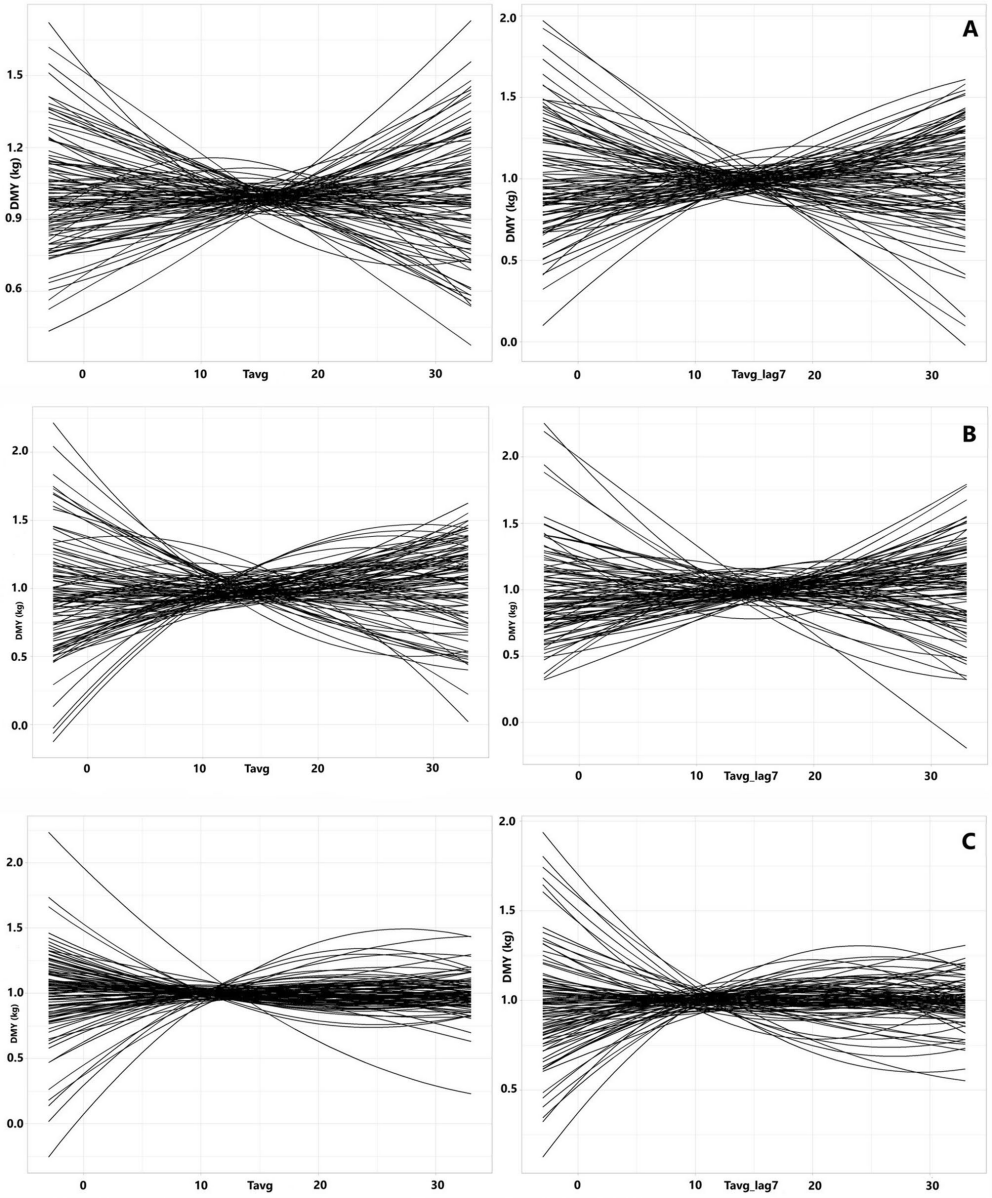
Individual animal reaction norms by lambing season (n = 100). Each curve represents the individual animal changes in daily milk yield (DMY, kg) in response to average air temperature variation on the milk test date (Tavg, °C) and week average air temperature preceding the milk test date (Tavg_lag7, °C), respectively. *A:* autumn lambing, *B:* winter lambing, *C:* spring lambing.

Examples of individual animal reaction norms by lambing season are illustrated in Fig. 2. These slopes reflect deviations from the population curves shown in Fig. 1, and constitute individual animal resilience phenotypes in the three lambing seasons. Substantial phenotypic variability among animals was observed within each season, which was particularly noticeable at either end of the temperature range. Presence of phenotypic variation is a prerequisite for the development of practices to improve animal resilience to weather fluctuation as discussed in further detail below.

Descriptive statistics of seasonal performance resilience phenotypes of individual Chios sheep are shown in Table 2. These phenotypes represent milk yield change in response to temperature fluctuations at 10 and 25 °C. The latter were selected as indicative of cold and hot weather, respectively, based on Fig. 1. Positive values of individual slopes indicate that milk yield increases with increasing temperature at the corresponding temperature level and milk yield decreases with dropping temperatures. Indicatively, autumn lambing individuals followed this pattern throughout the temperature range (Table 2). The opposite is true for negative values, characteristically exhibited in the winter lambing individuals under heat stress. A slope value close to zero suggests that milk yield of these ewes is generally unaffected by temperature change.

**Table 2:** Descriptive statistics (mean and SD in parentheses) of seasonal animal resilience phenotypes at different temperature levels.

Phenotype	Autumn	Winter	Spring
Tavg10	0.010 (0.014)	0.014 (0.024)	− 0.008 (0.016)
Tavg25	0.010 (0.017)	− 0.005 (0.019)	0.007 (0.006)
Tavg10_lag7	0.011 (0.016)	0.012 (0.021)	0.003 (0.015)
Tavg25_lag7	0.007 (0.018)	− 0.011 (0.019)	0.006 (0.007)

Tavg10, Tavg25: milk yield change by 1 °C temperature change at 10 °C and 25 °C on the milk test date, respectively; Tavg10_lag7, Tavg25_lag7: milk yield change by 1 °C cumulative average air temperature change during the week preceding the milk test date.

### Genetic parameters of animal resilience phenotypes and lifetime milk yield

Genetic parameters were estimated for the performance resilience phenotypes derived under cold (10 °C) and hot (25 °C) weather conditions. Heritability estimates of animal resilience by calendar season of lambing are presented in Table 3 and were significantly greater than zero in almost all cases. The lowest heritability estimated for resilience of spring lambing animals under hot temperatures (h^2^ = 0.03, SE = 0.09) and the highest for autumn and winter lambing ewe resilience under hot temperatures (h^2^ = 0.17, SE = 0.02).

**Table 3:** Heritability estimates of seasonal animal resilience phenotypes and correlation with lifetime milk production at different temperature levels (standard errors in parentheses).

Resilience phenotype	Season	h^2^	r_P_	r_A_
Tavg10	Autumn	0.15 (0.02)*	− 0.49 (0.01)*	− 0.75 (0.05)*
	Winter	0.13 (0.01)*	− 0.30 (0.01)*	− 0.27 (0.07)*
	Spring	0.14 (0.03)*	0.69 (0.01)*	0.84 (0.05)*
Tavg10_lag7	Autumn	0.16 (0.02)*	− 0.29 (0.01)*	− 0.43 (0.06)*
	Winter	0.12 (0.01)*	− 0.21 (0.01)*	− 0.09 (0.07)
	Spring	0.09 (0.02)*	0.36 (0.01)*	0.71 (0.12)*
Tavg25	Autumn	0.17 (0.02)*	− 0.76 (0.00)*	− 0.89 (0.02)*
	Winter	0.16 (0.01)*	− 0.78 (0.00)*	− 0.85 (0.02)*
	Spring	0.03 (0.02)	− 0.35 (0.01)*	− 0.02 (0.21)
Tavg25_lag7	Autumn	0.17 (0.02)*	− 0.51 (0.01)*	− 0.71 (0.05)*
	Winter	0.17 (0.02)*	− 0.49 (0.01)*	− 0.55 (0.05)*
	Spring	0.12 (0.03)*	− 0.40 (0.01)*	− 0.52 (0.11)*

Tavg10, Tavg25: milk yield change by 1 °C temperature change at 10 °C and 25 °C on the milk test date, respectively; Tavg10_lag7, Tavg25_lag7: milk yield change by 1 °C cumulative average air temperature change during the week preceding the milk test date, h^2^ Heritability, r_P_ Phenotypic correlation with corresponding lifetime milk production, r_A_ Genetic correlation with corresponding lifetime milk production. Estimates significantly different from zero (P < 0.01) are indicated with an asterisk.

Phenotypic and genetic correlations of performance resilience with corresponding lifetime milk yield are also shown in Table 3 attesting to both positive and negative associations between production and resilience depending on the season. For example, a positive genetic correlation of 0.84 (SE = 0.05) was estimated between lifetime milk production and resilience of spring lambing animals under cold temperatures in contrast with winter (r_A_ = − 0.27, SE = 0.07) and autumn (r_A_ = − 0.75, SE = 0.05) lambing animals. These correlations were significantly (P < 0.05) negative in autumn and winter lambing ewes under hot temperatures in almost all cases but were practically zero for spring lambing animals.

Correlation estimates between animal performance resilience phenotypes derived in different seasons are given in Table 4. Differences were observed between seasonal resilience phenotypes under cold temperatures, exemplified by a negative genetic correlation of − 0.27 (SE = 0.13) between autumn and spring resilience, and a positive estimate of 0.51 (SE = 0.08) between autumn and winter animal resilience. As a general remark, performance resilience of spring lambing ewes was always negatively correlated with that in other seasons, in concordance with phenotypic results at population and individual level presented previously (Figs. 1 and 2).

**Table 4:** Correlation estimates between different seasonal animal resilience phenotypes (standard errors in parentheses).

Resilience phenotype	Seasons	r_P_	r_A_
Tavg10	A-S	− 0.09 (0.02)*	− 0.27 (0.13)*
	A-W	0.14 (0.01)*	0.51 (0.08)*
	W-S	− 0.04 (0.02)*	− 0.16 (0.12)
Tavg10_lag7	A-S	− 0.06 (0.02)*	− 0.09 (0.15)
	A-W	0.17 (0.01)*	0.58 (0.07)*
	W-S	− 0.05 (0.02)*	− 0.18 (0.15)
Tavg25	A-S	0.09 (0.02)*	0.86 (0.35)*
	A-W	0.36 (0.01)*	0.55 (0.06)*
	W-S	0.13 (0.02)*	0.65 (0.22)*
Tavg25_lag7	A-S	0.14 (0.02)*	0.43 (0.15)*
	A-W	0.37 (0.01)*	0.51 (0.06)*
	W-S	0.23 (0.01)*	0.59 (0.11)*

Tavg10, Tavg25: milk yield change by 1 °C temperature change at 10 °C and 25 °C on the milk test date, respectively; Tavg10_lag7, Tavg25_lag7: milk yield change by 1 °C cumulative average air temperature change during the week preceding the milk test date, A-S autumn-spring, A-W autumn–winter, W-S winter-spring, r_P_ Phenotypic correlation between different seasonal resilience phenotypes, r_A_ Genetic correlation between different seasonal resilience phenotypes. Estimates significantly different from zero (P < 0.01) are indicated with an asterisk.

Additionally, associations between resilience to weather change on the day of milk measurement and resilience to cumulative weather fluctuations in the preceding week are shown in Table 5. Based on the genetic correlation estimates, these two performance resilience phenotypes are mostly under the same genetic control except, despite the minor phenotypic differences presented earlier. Finally, the association between animal resilience to hot and cold weather conditions is summarised in Supplementary Table S1.

**Table 5:** Correlation estimates between daily and cumulative animal resilience phenotypes by lambing season (standard errors in parentheses).

Season	Phenotypes	r_P_	r_A_
Autumn	Tavg10-Tavg10_lag7	0.93 (0.00)*	0.97 (0.01)*
	Tavg25-Tavg25_lag7	0.98 (0.00)*	0.98 (0.00)*
Winter	Tavg10-Tavg10_lag7	0.90 (0.00)*	0.93 (0.01)*
	Tavg25-Tavg25_lag7	0.97 (0.00)*	0.98 (0.00)*
Spring	Tavg10-Tavg10_lag7	0.82 (0.00)*	0.82 (0.05)*
	Tavg25-Tavg25_lag7	0.78 (0.01)*	0.42 (0.17)

Tavg10, Tavg25: milk yield change by 1 °C temperature change at 10 °C and 25 °C on the milk test date, respectively; Tavg10_lag7, Tavg25_lag7: milk yield change by 1 °C cumulative average air temperature change during the week preceding the milk test date, r_P_ Phenotypic correlation between different seasonal resilience phenotypes, r_A_ Genetic correlation between different seasonal resilience phenotypes. Estimates significantly different from zero (P < 0.01) are indicated with an asterisk.

## Discussion

The present study set out to generate new insights into the seasonality of performance resilience to weather fluctuations in livestock, using milk performance records of dairy sheep lambing in different calendar seasons, and corresponding meteorological data. Novel animal phenotypes were derived reflecting response of milk production to changing weather and substantial differences were observed across different seasons. For example, resilience of autumn lambing ewes in hot conditions reflected an increasing milk yield in contrast with a declined performance of winter lambing animals (Fig. 1). Autumn lambing animals are exposed to high summer temperatures towards the end of the lactation period when daily milk yield naturally declines. Therefore, they may develop a less severe l thermal load due to lower metabolic heat production^10^, which possibly renders them less vulnerable to heat stress at this point of their lactation. On the contrary, winter lambing animals are still in mid-lactation during the hot summer months, potentially resulting in higher metabolic stress which prevents the expression of their full milk yield potential^10^. Additionally, winter lambing animals seem to follow the *specialist-generalist* profile as described in terms of thermal adaptation^36,37^, characterised by maximal performance within the comfort temperature zone and diminishing performance at either colder or hotter weather conditions.

Animals lambing during the spring season seemed to be able to perform relatively better under heat stress temperatures. Their performance response to temperature fluctuation on the date or during the week preceding the milk test was positive as air temperature increased (Fig. 1). The continuous exposure of spring lambing Chios sheep to high summer temperatures during their lactation period may suggest a seasonal adaptation to these climate conditions^10^. Additionally, average milk yield following spring lambings is usually lower compared to autumn and winter lambings. Consistently with these results, low level of milk production has been previously reported for locally adapted sheep breeds, which favour fat deposition and body condition over milk production when improved feeding is provided^10,38^. On the other hand, the overall lower milk yield of the spring lambing animals might also be due to management effects, where farmers try to reduce feeding costs during summer months through free grazing of early summer harvested crops such as wheat and barley. Still, reduced milk production favours energy partitioning towards animal fitness needs, as manifested here by improved performance resilience. Furthermore, ewes that lamb and are milked in the period of long daylight duration tend to exhibit increased prolactin secretion during the milking months, which is a photoperiod sensitive hormone regulating the onset and continuation of the lactation process^39^. On the contrary, sheep starting their lactation during a period of shortening days have been shown to yield less milk compared to ewes milked during the long daylight period^40^. Accordingly, spring lambing sheep may benefit from long daylight throughout their lactation period and continue expressing their milk production potential despite high air temperatures.

Distinct resilience phenotypes were derived at individual level to reflect milk yield changes under cold (10 °C) and hot (25 °C) weather conditions and were treated as different animal traits. The temperature value reflecting hot conditions was based on the observed decline in milk yield in our data and was consistent with previously reported heat stress thresholds (above 20–25 °C) for Mediterranean dairy sheep^28,41^. Contrary to heat stress, cold stress has not been studied extensively in the Mediterranean region. Peana et al.^42^ were the first to report a substantial decline of milk yield below 15 °C in Sardinian dairy sheep. More recently, Ramón et al.^28^ estimated cold stress thresholds for different climatic variables, considering a critical point of milk yield changes due to daily average temperature at 11.5 °C for Mediterranean dairy sheep. Both studies corroborate our choice of temperature to represent cold stress.

Strong positive genetic correlation estimates between resilience of performance following autumn and winter lambings under cold and hot conditions reported here suggest that the two traits are, at least partially, under the same genetic control. For resilience under hot weather conditions, this was actually true for all three lambing seasons. However, milk performance resilience to cold weather following spring lambings behaved differently, manifested by nearly zero genetic correlations with the other two seasons. The latter may be largely attributable to the very few milk records available under low temperatures for the spring lambing ewes; therefore, this result should be viewed with caution.

Separate resilience phenotypes were also derived according to the duration of animal exposure to weather conditions. Thus, one phenotype was based on the air temperature on the same day as milk recording and another on the average air temperature during the week preceding the day of milk recording. The former is considered as resilience to weather conditions prevailing on the actual day of performance, whereas the latter is resilience to a cumulative weather effect of the entire past week. Differences were observed between these two resilience traits at the phenotypic level. There were also some differences in the association of the two traits with lifetime milk production. These differences may be attributed to the lactogenesis process taking place during the week preceding the actual milk production. Previous studies on the role of the prolactin hormone, which is essential for lactogenesis, have proven the interplay between decreased secretion of prolactin and heat stress in cattle^43–45^. Nevertheless, in most cases the genetic correlation between the two resilience phenotypes was very high, implying the same genetic control of daily and week-cumulative resilience. The exception was performance resilience following spring lambing under hot weather conditions, where the genetic correlation was moderately positive, implying a partially different genetic mechanism dictating how animals respond to heat stress on the day of milk measurement compared to thermal stress accumulating over a period of time. Further research at the genomic and transcriptional level may shed more light into the genetic architecture of the trait.

The individual animal resilience phenotypes developed in the present study exhibited significant phenotypic variability across all seasons. Different animals performed differently to temperature fluctuations under cold or hot conditions, consistently with previous research findings^37^. Additionally, we observed several stable milk producing animals independently of temperature change across all seasons, representing individuals whose milk production is minimally affected by weather volatility. Resilience phenotypes of these individuals emanated from reaction norm curves that were close to zero. Such animals could be characterised as the most resilient to weather fluctuation and their phenotypes could potentially constitute the desirable direction of selective breeding, if the latter were deemed feasible.

Indeed, the resilience phenotypes derived here exhibited significant genetic variation and heritability, implying that: (i) there are genetic differences among individual animals in their inherent capacity to respond to weather fluctuations and (ii) performance resilience is a trait that can be altered with genetic selection. Heritability estimates ranged between 0.03 and 0.17, depending on the lambing season and temperature level. Previous studies reported similar heritability estimates on other fitness-related traits in dairy cattle (h^2^ = 0.10–0.35)^4,26^, pigs (h^2^ = 0.15)^17^ and dairy goats (h^2^ = 0.21–0.30)^46^. Heritability estimates were highest for autumn lambings and lowest for spring lambings, implying a stronger environmental component for the latter, which may indicate a seasonal adaptation of these animals. Serradilla et al.^46^ reported also diminished heritability of milk traits while the heat load increased in different dairy goat breeds. Although relatively modest, heritability estimates of these traits are statistically significant and enable genetic improvement via selective breeding in a multi-trait context in order to mitigate against future changes in climate and weather variability^17^.

The next step would then be to determine the desirable direction of genetic selection for enhanced resilience and the genetic correlation with other traits in the breeding goal. As mentioned above, we reason that the desirable direction would be towards a slope of zero, implying no changes in milk production caused by weather fluctuation. This would require increasing the average values of performance resilience following winter and spring lambings under hot and cold weather conditions, respectively, and decreasing the mean for all other resilience phenotypes. In this context, the genetic correlation of resilience with milk production, which constitutes the primary breeding goal trait in the current genetic improvement programme of the Chios sheep, can be viewed as either antagonistic or favourable. Considering the above, genetic correlation of the resilience phenotypes with lifetime milk production in the low temperatures could be characterised as favourable across all lambing seasons; an increase in milk production, which is a wanted outcome of selection, would be always associated with a change in average resilience towards zero, which is also desirable. Similarly, a desirable genetic correlation could be assumed between resilience under hot conditions and milk production following autumn and spring lambings. Conversely, an antagonistic genetic correlation was found between resilience under hot temperatures and milk performance for winter lambings, consistent with the genetic antagonism between milk production and resilience as a fitness trait reported in previous studies on other ruminant species^29–31^. The antagonism between fitness and production has been extensively reported in Mediterranean dairy sector^5,26,27^ and it has been interpreted by the resource allocation patterns^47^, where high yielding animals allocate energy to milk production at the expense of increased environmental sensitivity^48^. Additionally, a zero genetic correlation was observed between resilience under high daily average temperatures and lifetime milk production following spring lambings, suggesting a possible seasonal adaptation of these ewes. The contrasting observations resulting from the above correlation estimates would suggest the need to develop separate customised breeding goals according to calendar season of lambing. In all cases, breeding goals will also need to consider the genetic correlation between performance resilience to cold and hot weather conditions, which differed by season of lambing in the present study. Correlations of resilience with other breeding goal traits including health and fertility should be also factored in. An economic analysis of all traits would be needed to determine the relative emphasis placed on each phenotype in the selective breeding programme.

As previously mentioned, the only weather variable exhibiting substantial variation in the geographic region of the present study was air temperature. Therefore, the study of performance resilience to air temperature change became our main scope. Admittedly, all results derived here are directly relevant to the studied breed and the prevailing climatic conditions in the region these sheep are raised. Future studies should consider the specificities of the animal populations in question and the appropriate environmental variables in the corresponding geographic regions and systems of production.

The present study was based on field data collected within the routine monthly recording process run by the Chios Sheep Breeders’ Cooperative ‘Macedonia’. In this regard, primary data are subjected to rigorous quality assurance protocols and biologically implausible entries are removed. Further edits were applied here to render the data suitable for the present study. Arguably, more frequent recording could facilitate the separation of weather from other environmental factors affecting animal performance. Nevertheless, use of widely accepted monthly recording has been proven an affordable data collection strategy for the study of animal resilience to weather fluctuations in genetic improvement programmes^49^.

To our knowledge, this is the first study on the seasonality of performance resilience in farm animals, where seasonal resilience phenotypes are developed and evaluated. Using population data on sheep milk performance and meteorological records on fluctuating Mediterranean weather conditions allowed us to expand on previous studies^4,29^ and contribute in understanding better the variability in animal resilience across distinct calendar seasons at population and individual level. Differences revealed by the genetic analyses between seasonal resilience traits indicate possible seasonal adaptation to climate according to calendar season of lambing within the studied population. Future studies on the genomic and transcriptional profile of these novel traits will shed light into the molecular architecture of resilience. The same methods applied here to assess performance resilience could be extended to functional and fitness-related animal traits, reflecting changes in feed intake, reproductive potential, disease susceptibility and mortality rate in response to fluctuating external climate and other environmental conditions. Awareness of the animal genetic profile regarding the physiological mechanisms involved in local and seasonal adaptation processes would be a crucial step towards coping with future challenges.

## Materials and methods

### Animal data

Individual milk yield records of purebred Chios sheep were obtained from the Chios Sheep Breeders’ Cooperative Macedonia. Data were from a total of 44,809 milking ewes raised in 145 flocks pertaining to the period 2003–2018. The farm management system in these flocks is characterised as semi-intensive. Ewes normally lamb (meaning they give birth) from September to May and their lambs suckle during the first 42 days post-partum before twice-a-day milking commences. The period of milking, known as lactation period, lasts normally 5–6 months. Flocks participate in the official milk recording scheme run by the Cooperative, according to which the daily milk yield is recorded on each ewe on a monthly basis following the A4 official method of the International Committee for Animal Recording^50^, as implemented routinely in the Chios breed^51^. Hereafter, these records will be referred to as test-day milk yield records.

Data edits removed records obtained less than 42 days after lambing (birth of lambs) and extreme values exceeding four standard deviations from the mean of the respective month of the ensuing lactation. Animals with less than three milk records during their productive life were excluded, too, resulting in an edited dataset consisting of 420,534 records by 36,908 animals.

Animal pedigree was also extracted from the Cooperative’s database including 101,493 animals, 2324 sires and 26,008 dams.

### Weather data

Weather data were obtained from the Hellenic National Meteorological Service for 14 weather stations close to the corresponding farms of the study. These data included average daily air temperature and relative humidity. These two variables were combined to develop a Temperature Humidity Index (THI), calculated using the following formula^5^:

1 $$THI=T-\left(0.55*\left(1-RH/100\right)\right)*\left(T-14.4\right)$$

where THI is the temperature-humidity index, T and RH represent the average daily temperature (°C) and humidity (%) respectively.

Weather variables from the closest meteorological station were matched to the respective test-day milk yield records for the study of the weather effect on milk production on the day of measurement. Furthermore, the same weather variables were averaged over the week preceding the test-day of milk recording and matched with the respective records in order to study the cumulative effect of weather on milk yield.

### Development of performance resilience phenotypes

Reaction norm functions were fitted to the following random regression models in order to derive animal performance resilience phenotypes reflecting changes in milk yield in response to weather variability:

$${Y}_{ij}= X + f\left(\beta , {X}_{j}\right)+ {a}_{i}+{e}_{ij}$$

$${Y}_{ij}= X + f\left(\beta , {X}_{j}\right)+ {f}_{i}\left({a}_{i}, {X}_{j}\right)+{e}_{ij}$$

where Eq. (2) describes the population level response and Eq. (3) the individual animal response. *Y*_*ij*_ corresponds to test-day (24-h) milk yield of individual animal *i* under weather variable (air temperature or THI) *j*, *X* represents a set of fixed effects on test-day milk yield, *f(β, X*_*j*_*)* represents the population reaction norm function using a Legendre polynomial of second degree and describing the relationship between the average animal performance and weather variable value *j*, *a*_*i*_ corresponds to the individual animal effect *i*, *f(a*_*i*_*, X*_*j*_*)* represents the individual reaction norm function using a Legendre polynomial of second degree, describing the relationship between individual animal *i* and weather variable value *j* (expressed as a deviation from the population reaction norm) and *e*_*ij*_ corresponds to the residual.

Following preliminary statistical analysis, the following fixed effects were included in the above models: farm, lactation number, lambing year and month, and number of days from lambing. BLUPF90 software^52^ was implemented to run the analyses in R studio with R.3.6.1^53^.

The individual animal performance resilience phenotypes were based on the slopes of the individual reaction norms, estimated as the derivatives corresponding to different values of the weather variable on the individual response curve. These values reflect milk yield change across the respective weather variable gradient. For example, resilience phenotypes would describe the change in milk yield by 1 °C temperature change at a certain temperature level. The latter was set to 10 °C and 25 °C to represent distinct resilience traits under cold and heat stress temperatures, respectively, in the ensuing analyses.

In order to assess the seasonal impact on animal performance resilience, separate resilience phenotypes were developed and examined by three calendar seasons of lambing defined as autumn (lambings in September–November), winter (December-February) and spring (March–May). Total lifetime milk yield per animal and lambing season was calculated from the test-day milk records following the rules of the International Committee for Animal Recording (ICAR, 2020)^54^. Animals with multiple lambings could have had records in different seasons.

At the end of the process, individual animal performance resilience phenotypes were available for the two above mentioned temperatures (10 °C and 25 °C) and the three calendar seasons of lambing studied as distinct resilience traits.

### Genetic parameters of animal performance resilience phenotypes and lifetime milk yield

Trait heritability and between trait correlation estimates were derived from univariate and bivariate statistical analyses using mixed models. Fixed effects included farm, first lambing year and month, total number of lactations, and total number of milking days. Individual animal was fitted as a random additive genetic effect including all pedigree data available. Genetic and phenotypic correlations were estimated among the three seasonal resilience phenotypes, and between resilience and lifetime milk yield within calendar season. These analyses were performed using the ASReml software^55^. Statistical significance of all estimates was assessed using the two-tailed Student’s t-distribution. Significance of variance components was also evaluated using likelihood ratio test, based on the comparison of the log likelihoods of the model with and without the random animal genetic effect.

## Supplementary Information

Supplementary Information.

## Data Availability

Data analysed during the current study are available from the corresponding author on reasonable request. Data generated are available at https://data.mendeley.com/datasets/34gn8ddk43/1.
