# Supplementary material for: Understanding the seasonality of performance resilience to climate volatility in Mediterranean dairy sheep
Source: Sci Rep. 2021 Jan 21;11:1889. doi: 10.1038/s41598-021-81461-8 (PMC7820498; doi:10.1038/s41598-021-81461-8)
Supplement: Supplementary file 1 — Supplementary Information. [file 41598_2021_81461_MOESM1_ESM.pdf]

Understanding the seasonality of performance resilience to climate volatility in Mediterranean dairy sheep

Authors & Affiliations:

Valentina Tsartsianidou[1*], Vanessa Varvara Kapsona[2], Enrique Sánchez-Molano[3], Zoitsa Basdagianni[4], Maria Jesús Carabaño[5], Dimitrios Chatziplis[6], Georgios Arsenos[7], Alexandros Triantafyllidis[1] & Georgios Banos[2,7]

[1] Department of Genetics, Development & Molecular Biology, School of Biology, Aristotle University of Thessaloniki, 54124 Thessaloniki, Greece

[2] Department of Animal and Veterinary Sciences, Scotland's Rural College, Roslin Institute Building, Easter Bush, Midlothian EH25 9RG, UK

[3] Division of Genetics and Genomics, The Roslin Institute and Royal (Dick) School of Veterinary Studies, University of Edinburgh, Easter Bush, Midlothian EH25 9RG, UK

[4] Department of Animal Production, School of Agriculture, Aristotle University of Thessaloniki, 54124 Thessaloniki, Greece

[5] Departamento de Mejora Genética Animal, Instituto Nacional de Investigación y Tecnología Agraria y Alimentaria (INIA), 28040 Madrid, Spain

[6] Laboratory of Agrobiotechnology and Inspection of Agricultural Products, Department of Agriculture, International Hellenic University, Alexander Campus, 57400 Sindos, Greece

[7] Laboratory of Animal Husbandry, School of Veterinary Medicine, Aristotle University of Thessaloniki, 54124 Thessaloniki, Greece

*corresponding author:

tsarvale@bio.auth.gr

+306951626615

Supplementary Material

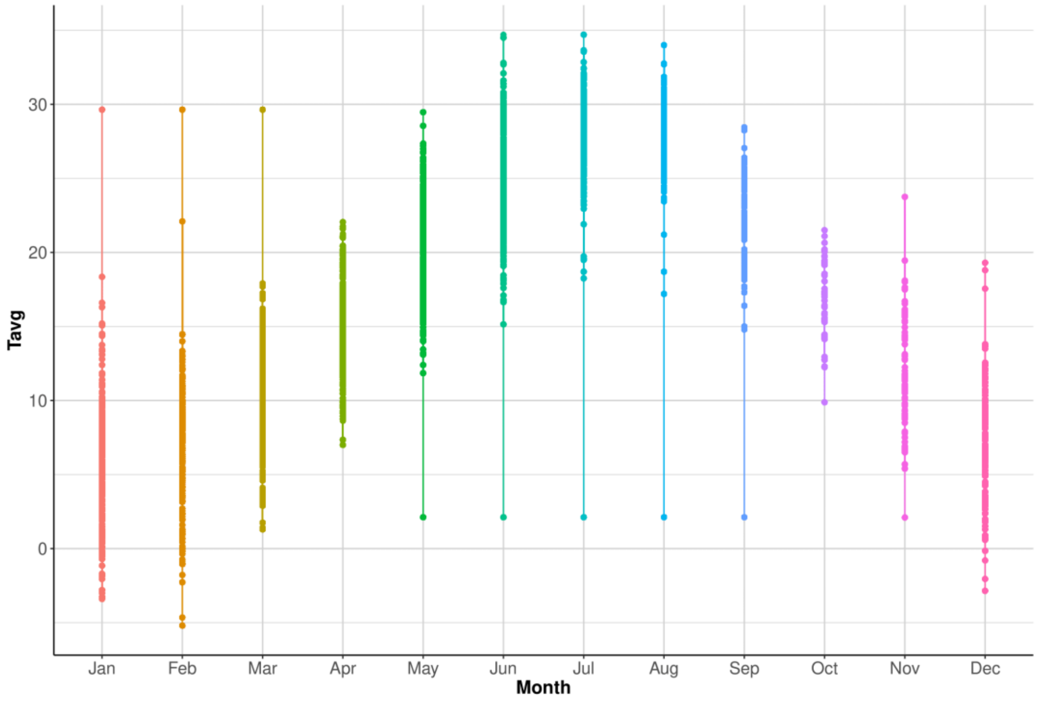

Figure S1 Average air temperature (Tavg) and dispersion by calendar month during the period of study (2003-2018).

Table S1: Correlation estimates between animal resilience to hot and cold weather by lambing season (standard errors in brackets). Tavg10, Tavg25, Tavg10_lag7, Tavg25_lag7: milk yield change by 1°C temperature change at 10°C and 25°C on the milk test date and cumulative average air temperature change during the week preceding the milk test date respectively. Estimates significantly different from zero (P < 0.01) are indicated with an asterisk.

Season	Phenotypes	r_P	r_A
Autumn	Tavg10-Tavg25	0.77 (0.00)*	0.86 (0.03)*
	Tavg10-Tavg25_lag7	0.63 (0.01)*	0.76 (0.04)*
Winter	Tavg10-Tavg25	0.48 (0.01)*	0.46 (0.05)*
	Tavg10-Tavg25_lag7	0.50 (0.01)*	0.52 (0.05)*
Spring	Tavg10-Tavg25	-0.03 (0.01)*	-0.22 (0.25)
	Tavg10-Tavg25_lag7	-0.19 (0.01)*	-0.73 (0.10)*
